# Plant Growth-Promoting Rhizobacteria Isolated from Degraded Habitat Enhance Drought Tolerance of Acacia (*Acacia abyssinica* Hochst. ex Benth.) Seedlings

**DOI:** 10.1155/2020/8897998

**Published:** 2020-10-29

**Authors:** Alemayehu Getahun, Diriba Muleta, Fassil Assefa, Solomon Kiros

**Affiliations:** ^1^College of Natural Sciences, Addis Ababa University, Addis Ababa, Ethiopia; ^2^Addis Ababa Institute of Technology, Addis Ababa University, Addis Ababa, Ethiopia

## Abstract

Drought stress (DS) is the most impacting global phenomenon affecting the ecological balance of a particular habitat. The search for potential plant growth-promoting rhizobacteria (PGPR) capable of enhancing plant tolerance to drought stress is needed. Thus, this study was initiated to evaluate the effect of inoculating *Acacia abyssinica* seedlings with PGPR isolated from rhizosphere soil of Ethiopia to enhance DS tolerance. The strains were selected based on *in vitro* assays associated with tolerance to drought and other beneficial traits such as salinity, acidity, temperature, heavy metal tolerances, biofilm formation, and exopolysaccharide (EPS) production. The strains with the best DS tolerance ability were selected for the greenhouse trials with acacia plants. The results indicate that out of 73 strains, 10 (14%) were completely tolerant to 40% polyethylene glycol. Moreover, 37% of the strains were strong biofilm producers, while 66 (90.41%) were EPS producers with a better production in the medium containing sucrose at 28 ± 2°C and pH 7 ± 0.2. Strains PS-16 and RS-79 showed tolerance to 11% NaCl. All the strains were able to grow in wider ranges of pH (4–10) and temperature (15–45°C) and had high tolerance to heavy metals. The inoculated bacterial strains significantly (*p* ≤ 0.05) increased root and shoot length and dry biomass of acacia plants. One of the strains identified as *P. fluorescens* strain FB-49 was outstanding in enhancing DS tolerance compared to the single inoculants and comparable to consortia. Stress-tolerant PGPR could be used to enhance acacia DS tolerance after testing other phytobeneficial traits.

## 1. Introduction

Nowadays, the world has been terrified by global climate change scenarios. The scarcity of water is amongst a problem seen in the world which will drive the severity of drought episodes [1]. Degrading environment, rising population, and increased demand for resources affect severely ecological stability [2]. Action is needed to face the global threats arising from the effects of climate change, which could increase episodes of drought, salinity, toxicity by heavy metals, soil acidity, and extreme temperatures [3]. Drought stress (DS) is the most impacting phenomena that affects ecological integrity and ultimately results in degraded habitats with poor and/or no productivity [4]. It is estimated that drought covers approximately 41% of earth's land surface [5] and threatens more than 50% of arable lands and causes a 50% loss in crop yields, social, economic crisis, and environmental impacts [6]. Drought increases the demand for irrigation, which already comprises 70% of global water consumption [7]. Plants have evolved different mechanisms to mitigate DS that include a series of molecular, cellular, and physiological adaptations [8, 9]. All DS-associated problems result in loss of soil microbial diversity, soil fertility, and aggravate competition for nutrients. These call for urgent intervention measures using drought-tolerant microbes as eco-friendly approaches [10]. Hence, integrating drought-tolerant beneficial microbes as a component of ecological systems to enhance plant drought tolerance might represent an interesting strategy. At the moment, efforts have been focused on harnessing the potential of phytobeneficial soil microbes to enhance environmental rehabilitation to combat the negative impacts of drought [11]. The positive influence of PGPR on conferring resistance to DS in many crops and trees has been reported [12]. Also, the production of biofilms and exopolysaccharides provides remarkable protection from external stress, decreases microbial competition, gives protecting effects to the host plants, and increases soil aggregation [13].

Numerous studies have documented the potential of many PGPR genera including *Klebsiella*, *Pseudomonas*, *Acinetobacter*, *Paenibacillus,* and *Bacillus* in enhancing plant stress tolerance in dryland areas [14, 15]. PGPR can enhance plant stress tolerance by an array of mechanisms that encompass the production of ACC deaminase [16], regulation of the hormonal balance of cytokinins [16], gibberellins [17], EPS [18], and microbial biofilm formation for protection from external stresses [13]. Screening for stress tolerance is an important parameter while selecting bacterial strains for the development of biofertilizers since the performance of PGPR is constrained by environmental stresses including temperature, desiccation, pH, alkalinity/acidity, and salinity in the soil [19]. Due to their multiple traits, the search for suitable and rhizosphere competent PGPR becomes interesting and can be used as inoculants for biofertilization and biocontrol purposes in agriculture, forestry, and environmental rehabilitation. Acacia is highly used for conserving and improving degraded soils and landscapes. *Acacia senegal* is a key component of traditional agroforestry and a valuable tree species for restoration of soil fertility [20]. It is also used as belt rehabilitation at Dilling area (South Kordofan) in solving the problems of the traditional agricultural limited land [21]. Therefore, the selection of stress-tolerant bacterial strains might be critical for improving the field performance of diverse crops including woody plants. Hence, this work aimed to identify and characterize PGPR isolates that could enhance stress tolerance by promoting the growth of acacia seedlings.

## 2. Materials and Methods

### 2.1. Rhizobacteria Growth Conditions and Identification

*Acacia* and *Juniperus* rhizosphere soil samples were collected from degraded soil of north Shewa Zone, Oromia National Regional State, Ethiopia. It is located at 9° 45′ 57″ N and 38° 42′ 06″ E, and the soil is characterized as sandy clay loam [22]. Soil samples were processed within 24 h, and a tenfold serial dilution was made using sterilized distilled water. Primary isolations were done on Nutrient and King's B agar (both from Himedia). From an appropriate dilution factor, 100 *μ*L of the suspension was plated on Nutrient and King's B agar and incubated at 28 ± 2°C for 24–48 h [23]. Tryptic Soy Agar (TSA, Himedia) was used for the screening purpose. Since water is the limiting factor in the study area, drought stress (DS) tolerance was taken as a baseline parameter and other plant growth-promoting (PGP) traits to select the potential strains were considered.

A total of 80 PGPR isolates with promising phytobeneficial traits and DS tolerance were selected. Since 7 isolates showed poor gel quality with very short base pair sequences, the number is further reduced to 73. Firstly, the bacterial DNA was extracted and isolated using the DNeasy Blood & Tissue kit (QIAGEN®, Germany). The 16S rRNA genes were amplified using universal primers fD1 (forward) and rD1 (reverse). Primers used for gene amplification had the following sequences: fD1 (5′-AGAGTTTGATCCTGGCTCAG-3′) and rD1 (5′-AAGGAGGTGATC CAGCC-3′) [24]. The PCR products were purified using PureLink® Quick PCR Purification Kit and separated in a 1.5% agarose gel to be examined under a UV illuminator (Locus Biotechnology L-Pix, Brazil) [25]. The PCR condition was set at initial denaturation at 95°C for 2 min, denaturation at 94°C for 15 sec, annealing at 55°C for 45 sec, elongation at 72°C for 2 min, and final elongation at 72°C for 5 min. Finally, the PCR products were eluted and sequenced using 3500XL Genetic Analyzer (Hitachi, Applied Biosystems, Londrina, Brazil) with the incorporation of dideoxynucleosides (dd NTPs) into the reaction mixture. The sequence was done in Brazil, Londrina, and the sequence data was edited with Bionumeric 3.2 version [26]. Sequences were further analyzed using BLAST software of the National Center of Biotechnology Information (NCBI) website. Phylogenetic analysis of partial 16S rRNA gene sequences was done using the Mega 7 software version 7.0.2 [27].

### 2.2. Screening Drought Stress (DS) Tolerance of PGPR

All the 80 isolates were tested for *in vitro* drought tolerance and plant growth-promoting traits. Osmotic stress was tested by adding 40% of polyethylene glycol-6000 (PEG) (400 g/L to Tryptic Soya Broth (TSB) g/L: pancreatic digest of casein 17; a peptic digest of soya bean meal 3; sodium chloride 5; dextrose 2.5; and dibasic potassium phosphate 2.5). A 1 mL of the bacterial culture at the concentration of 1 × 10^7^ CFU/mL was estimated by optical density (OD) at 600 nm to be used as initial inoculum and added to the test tubes containing 10 mL of TSB amended with PEG 6000 to adjust the osmotic pressure at 1.76 Mega Pascal (MPa). The inoculated tubes were incubated at 28 ± 2°C for 24 h, and OD was recorded after 3 days. The OD values of drought tolerance were determined as follows: completely sensitive OD < 0.3; sensitive OD = (0.3–0.39); tolerant OD = (0.4–0.5), and completely tolerant OD > 0.5 [28].

### 2.3. Qualitative Assay for Biofilm Detection

#### 2.3.1. Plate Method (PM)

Mucoid nature of the bacterial colonies was observed after growth on Congo red agar (CRA) medium composed of (g/L): brain heart infusion broth, 37; sucrose, 5, agar, 10; Congo red dye, 0.8 [29]. Eighteen-hour-old bacterial cultures were streaked on the CRA plates and incubated at 28 ± 2°C for 24–48 h and observed for colony color. Black colonies with a dry crystalline consistency indicate biofilm production [30].

#### 2.3.2. Tube Method (TM)

Biofilm formation ability was observed by its adherence capacity to the walls of culture tubes [30]. A loopfull of each bacterial strain grown on TSB plates for 24 h was inoculated into 10 mL of nutrient broth with different NaCl concentrations (100 and 150 mM which is used to show enhanced absorbance) in test tubes followed by shaking at 95 rpm for 24–48 h. The culture medium with bacteria was discarded, and the tubes were washed with 3 mL of 1X phosphate-buffered saline (PBS) of pH 7. A 3 mL of 2% crystal violet solution was added and left for 15 min. Tubes were then washed with sterile water and allowed to dry, and the tubes were visually observed for the presence of biofilms rings on the inner walls of the test tubes. Tubes then received 1.5 mL of 33% glacial acetic acid and mixed gently to measure OD at 570 nm. PBS served as control. Biofilm formation in tubes was detected when a visible film (ring) lined the wall and the bottom of the tubes [31].

### 2.4. Exopolysaccharide Production

The qualitative determination of exopolysaccharide production was performed according to Paulo et al. [32]. Discs of sterile filter paper (5 mm) which were inoculated with 4 *μ*L of each isolate placed in Petri dishes containing nutrient agar medium g/L: (peptone 5; sodium chloride 5; beef extract 1.5; yeast extract 1.5 and agar 15) for the production of EPS test. This was evaluated by the size of the halo produced with its slime appearance. The production of EPS was confirmed by mixing a portion of the mucoid substance in 2 mL of chilled absolute ethanol, where the formation of a precipitate indicates the presence of EPS [32]. Similarly, each isolate was cultured at 28 ± 2°C but varying the type of sugar (sucrose, glucose or lactose) each at 10 g/L concentrations and the pH (5.5 ± 0.2 and 7.5 ± 0.2). After 48 h of growth, EPS production was evaluated based on the mucoid nature of growth around 5 mm discs.

### 2.5. Growth and Ecophysiological Characterization

For each biochemical and physiological tests, growth was determined by reading OD at 600 nm in nutrient broth (g/L): peptone, 5; NaCl, 5; beef extract, 1.5; yeast extract, 1.5. In all cases of tolerance measurements, the viability of the isolates was checked by streaking on TSA immediately following the OD reading and incubated at 28 ± 2°C for 24 to 48 days to confirm bacterial growth [33].

#### 2.5.1. Salt, pH, Temperature, and Heavy Metal Tolerance

Tolerance to salinity was evaluated on TSA medium containing 1%, 3%, 5%, 7%, 9%, and 11% (w/v) NaCl [34]. pH tolerance was tested in nutrient broth by adjusting the pH to 4, 5, 6, 8, and 10 with either 1N NaOH or HCl [35]. Temperature tolerance was evaluated by growing bacterial cultures in TSA at 4°C, 15°C, 25°C, 35°C, 40°C, 45°C, and 50°C. The agar dilution method was used to test the heavy metal (HM) tolerance of PGPR isolates [36]. A loopfull of 24-hour-old bacterial culture grown in the nutrient broth was streaked on Mueller-Hinton Agar (MHA) [37] plates amended with increasing concentrations (50, 100, and 300 *μ*g/mL) of different heavy metals (lead from (Pb(CH_3_COO)_2_.3H_2_O, zinc from ZnSO_4_.5H_2_O, copper from CuCl_2_.2H_2_O, manganese from MnSO_4_.4H_2_O, and iron from FeSO_4_.6H_2_O). Plates were incubated at 28 ± 2°C for 24 h and examined visually for the presence or absence of growth where the presence of growth was recorded as resistance/tolerance (R) and the absence of growth was recorded as susceptible (S). Unamended Mueller-Hinton Agar plates were used as controls to evaluate tolerance [38].

### 2.6. Greenhouse Experimental Trials

Pot trials were performed under greenhouse conditions at the Department of Microbial, Cellular, and Molecular Biology, Addis Ababa University. It was done to evaluate the potential of PGPR strains based on phytobeneficial traits exhibited for drought stress experiments using *A*. *abyssinica* plants. The seeds were collected from the highland region of Ethiopia and were scarified with concentrated H_2_SO_4_ in flasks to break seed dormancy. The flasks were swirled occasionally over 25–35 minutes [39]. Eight seeds were kept in equidistance position in sterilized Petri plates containing sterile moist filter paper and cotton for 7 days for germination [40]. After seeds germinated, four seedlings were transplanted into sterile plastic pots (20 × 15 cm) filled with 3 kg sandy clay loam soil autoclaved for 1 h. Plants were kept in well-watered conditions and fertilized with half-strength Hoagland solution each week to obtain nutrients at a free access rate for 60 days [41]. Plants were inoculated during transplanting and 7 days intervals after transplantation with 15 mL test strains (10^8^ CFU/mL) for 60 days. After 60 days of growth, plants were regularly watered to maintain 20% moisture by measuring the weight of pots every two days. The experiment was performed with a completely randomized design (CRD) and replicated three times. Plants were harvested after 2 weeks of water suppression, and data on root length, shoot length, and root and shoot dry biomass were recorded [42].

### 2.7. Data Analysis

Analysis of variance (ANOVA) was used to test for significant differences of measurements of each bioassay, whereas Duncan's multiple range test (DMRT) was employed to show significant differences among diverse treatments (mean separation) at *p* ≤ 0.05. Values are presented as mean ± standard deviation (SD). All the statistical analyses were performed using the Statistical Analysis System (SAS) version 9.0 software package [27]. All phylogenetic analyses were performed with the software MEGA 7 [43].

## 3. Results

### 3.1. Microorganism Isolation and Identification

**Figure 1:**
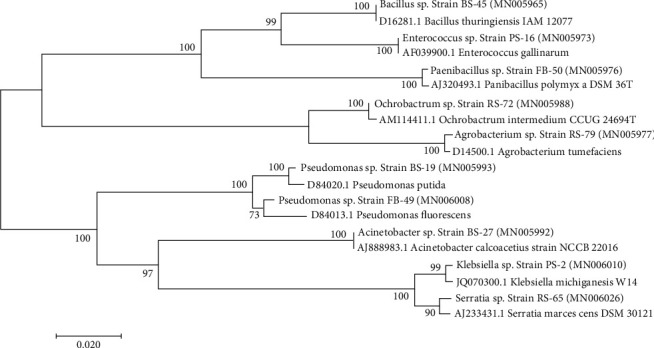
The phylogenetic relationship of top ten drought-tolerant strains with closest species. Accession numbers are indicated in brackets with bold text.

Eighty rhizobacterial strains were isolated from highly degraded soil of Ethiopia. But, 7 isolates were never sequenced, and 73 isolates were used for our purposes. Twenty-two isolates showed supreme drought tolerance. Of these, 10 completely drought-tolerant (CT) strains were used for plant growth promotion experiments in the greenhouse. The relationship among them with the closest species is shown in Figure 1 and Table 1.

### 3.2. Tolerance of Rhizobacteria to Stresses

#### 3.2.1. Drought Stress

**Figure 2:**
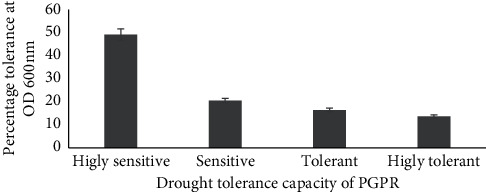
The percentage of drought tolerance classification of PGPR recovered from degraded soil.

From the 73 tested bacterial isolates, 10 (14%) were categorized as completely tolerant (CT) to DS with OD > 0.5 followed by 12 (16%) in the class of tolerant (T) with OD that ranged from 0.40 to 0.47 and 15 (21%) as sensitive (S) with OD 0.3 to 0.36, and the remaining 36 (49%) grouped as completely sensitive (CS) with OD that ranged from 0.06 to 0.25 (Figure 2).

Out of 73, 10 isolates showing completely drought tolerant (CT), and multiple PGP traits were selected for other stress assays and greenhouse experiments. The mean drought tolerance of each isolate is shown in Table 2. The highest drought tolerance (0.64) OD value was observed in *P. fluorescens* strain FB-49 followed by *P. putida* strain FB-49 with OD value 0.60.

### 3.3. Qualitative Biofilm Detection

**Figure 3:**
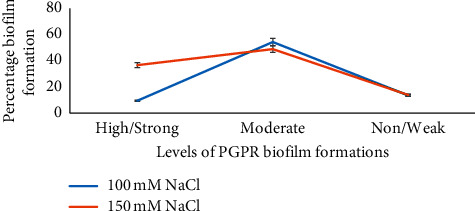
Classification and comparisons of bacterial biofilm formation abilities at 100 mM and NaCl 150 mM NaCl concentrations.

Most of the isolates produced black colonies on CRA after 24–48 h (Supplementary Figure S1). In the tube method, the formation of visible thick film inside the wall of tubes and their bottom (supplementary Figure S2). Out of 73, 27 (37%) isolates were strong biofilm producers, while 49% were moderate, and the remaining (14%) were weak or nonbiofilm producers using test tubes method (TM) at 150 mM NaCl. By using TM but with different NaCl concentrations (100 mM), 10%, 55%, and 34% were perceived as strong, moderate, and weak biofilm-producing PGPR isolates, respectively (Figure 3).

**Figure 4:**
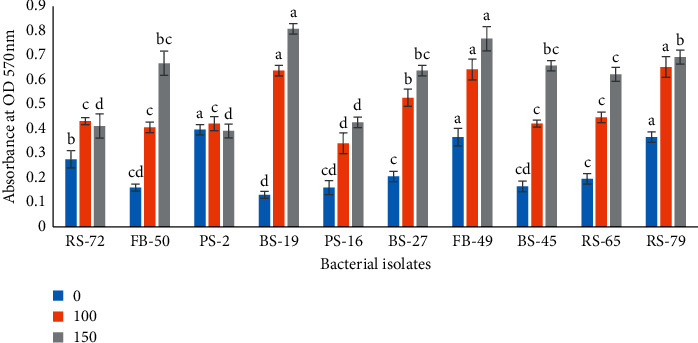
The optical density (OD at 570 nm) as the measure of the activity of biofilm formation for PGPR isolates with three NaCl (0, 100, and 150 mM) concentrations.

The results indicated that the activity of biofilm formation was increased with increasing NaCl concentration. The highest significant increase was recorded in BS-19 (0.805 OD) and FB-49 (0.765 OD) isolates treated with 150 mM NaCl, while the lowest was observed in isolate PS-2 (0.39 OD) (Figure 4).

### 3.4. Exopolysaccharide Production

The production of EPS was determined based on both mucoid colony production on culture medium and precipitate formation in chilled ethanol in a test tube (Supplementary Figures S3). Of the 73 PGPRs evaluated for the EPS production, 66 (90.41%) were positive, while the remaining 7(9.59%) were negative. The medium containing sucrose induced a higher number of isolates to produce EPS, with 41 (48.2%) positive results, most of which (65.85%) were at 28 ± 2°C and pH 7 ± 0.2. The second-highest number of EPS-producing isolates was found in the medium containing glucose, with 26 (30.58%) positive results, of which 61.53% were under the same conditions of pH and temperature that tested best with sucrose. Moreover, the medium containing lactose resulted in 18 (21.17%) positive results, with 61%, also under the same conditions of pH and temperature of other media (data not shown).

### 3.5. Salt, pH, Temperature, and Heavy Metal Tolerance

#### 3.5.1. Tolerance at Different NaCl Concentrations

**Figure 5:**
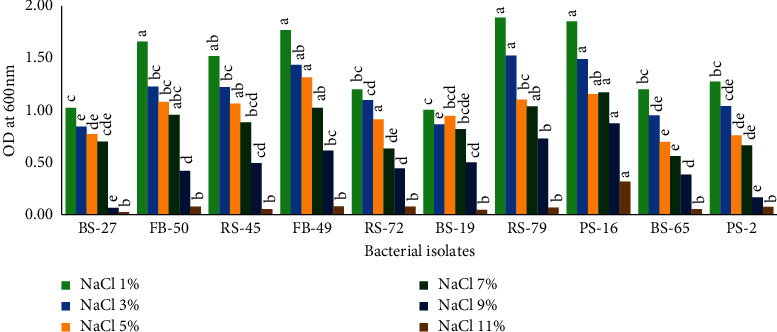
Effect of NaCl concentration on the growth of ten selected PGPR strains. Bars represent mean ± SD of three replicates. Treatments followed by different letters indicate significant difference over control using Duncan's multiple range test (*p* ≤ 0.05) (*n* = 3).

Our results indicate that PGPR strains could grow over a wide range of NaCl (1 to 11%) concentrations (Figure 5). Isolates PS-16 and RS-79 showed the highest NaCl tolerance followed by FB-49 and FB-50 with 9%. BS-19 was identified as the least tolerant. However, higher NaCl concentration led to a drastic reduction in the growth of bacterial isolates.

#### 3.5.2. Growth at Different pH Ranges

In our study, a wide pH range tolerance was confirmed in PGPR ability to survive both in acidic and alkaline soils. Among different levels of pH tested, all the isolates showed maximum growth at pH 7 followed by pH 10 and pH 9, while the minimum growth of most of the bacterial isolates was observed at pH 4. Isolates RS-79, FB-49, BS-65, and PS-2 grew better on agar medium of pH 5 and FB-49 and RS-79 at pH 4 (Table 3).

#### 3.5.3. Response to Different Temperatures

All the 73 isolates were able to grow within a broad range of temperature (20°C to 45°C) but grew not at 4°C or 50°C as confirmed on solid agar medium. Maximum growth was achieved at 25°C, 35°C, and 40°C but lower at 15°C and 45°C (Table 4). Four (FB-50, RS-45, FB-49, and RS-79) isolates exhibited remarkable tolerance to high temperature (45°C) followed by BS-19.

#### 3.5.4. Tolerance of PGPR to Heavy Metals (HMs)

**Figure 6:**
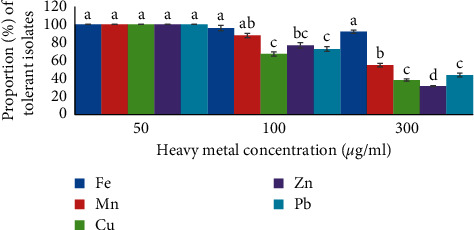
Tolerance of PGPR isolates to HM concentration. The different letters on the standard error (SD) bars indicate a significant difference using Duncan's multiple range test at *p* ≤ 0.05. Values are means of three of replicates.

Many isolates were tolerant to various concentrations of heavy metals tested (Figure 6). All isolates (100%) showed resistance to 50 *μ*g/mL of Fe, Mn, Cu, Zn, and Pb, whereas almost all isolates were able to grow on the medium containing 100 *μ*g/mL of Fe and Mn. However, fewer isolates grew on the medium containing the same concentration of Zn (77% of the isolates), Pb (73%), and Cu (67%), respectively. Moreover, bacteria growth significantly declined (*p* ≤ 0.05) to 32%–44 at 300 *μ*g/mL increase in concentrations of the heavy metals, except Fe (Table 5).

### 3.6. Drought Stress Enhancements in Acacia under Greenhouse Trial

**Figure 7:**
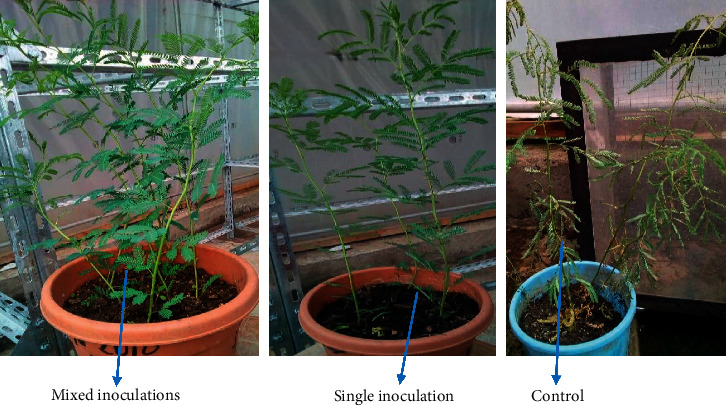
Drought stress enhancements of PGPR isolates in *Acacia* plants. Mixed = FB-50 + BS-27 + BS-19 + FB-49 + D; single = FB-49 + D; and control = without inoculation + D.

Inoculation with bacterial consortia had a significant (*p* ≤ 0.05) effect on plant biomass 57.3 cm, 19.3 cm, 2.1 g, 0.8 g, and 16.7 in SH, RL, SDW, and RDW and number of leaves per plant, respectively, compared to noninoculated control. Among single inoculants, *P. fluorescens* and *P. polymyxa* showed the maximum *A. abyssinica* performance under drought conditions. *Klebsiella michiganensis* showed the least drought stress improvement (38.7 cm) in SH compared to the other singly inoculated plants but performed better compared to noninoculated control treatment (Table 6). The performance of acacia in the greenhouse trials is shown in Figure 7.

## 4. Discussion

In the present study, effective PGPR isolates that grew in medium with reduced water content is considered as drought tolerant. Because 30% of the bacterial isolates showed *in vitro* tolerance to 40% PEG. The tolerance is mainly associated with biofilm formation and EPS production potential of the isolates. This is an initial selection of bacterial isolates based on their ability to grow in medium and is an interesting approach for other stress-associated assays. This feature is very crucial for degraded land restoration under water-stressed conditions by inoculating such potential isolates. The variation in drought tolerance among bacteria may be related to specific adaptations and gained strain-specific traits. The bacteria reaching OD greater than 0.5 categorized as completely drought tolerant [28]. Also, drought-tolerant *Rhizobium* sp. survived with 45% of PEG-enhanced drought tolerance of sesbania [44]. In another trial, *Pseudomonas* spp. and *Bacillus* spp. with good plant growth-promoting ability survived 40.5% PEG concentration [45]. The mechanisms by which PGPR can survive and adapt to extreme drought conditions are associated with secretion of EPS [46, 47], biofilm formation [48, 49], and ACC (1-aminocyclopropane-1-carboxylate deaminase) production [47, 50] and induced systemic tolerance by bacterial compounds [51] and other phytobeneficial traits. Moreover, drought tolerance occurs on wheat root colonization with *Paenibacillus* spp. and *Bacillus* spp. that can boost plant survival under drought stress [52, 53].

In the present study, we observed a dramatic change in PGPR ability to form a biofilm in 100 and 150 mM NaCl concentration. The current finding indicates that the activity of biofilm formation was increased with increasing NaCl concentration. The formation of biofilm and exopolysaccharide kept the viability of bacterial cells under salt stress to protect them in the rhizosphere. This is due the higher ionic strengths that are known to reduce the repulsion between a bacterial cell and a material surface. Moreover, it is well known that salt stress induces biofilm formation [54]. Biofilm-producing cells are attached to biotic or abiotic surfaces since biofilms provide important environmental reservoirs and protection for bacteria [55]. Biofilm assists drought tolerance by producing extracellular matrices to maintain a hydrated root environment, increasing root-adhering soil and stability [49]. Biofilms contain sugars and oligo- and polysaccharides that can play various roles in bacteria-plant interactions, e.g., in improving water availability in the root medium. Several studies have shown that different chemical substances or physical parameters affect the biofilm expression such as NaCl concentration and presence or absence of oxygen [29]. Besides, the process of biofilm is affected by several factors such as temperature, pH, nutrients content, salinity, contact surface properties, and microbial strains [56].

The present finding confirms that the best situation for EPS production by PGPR isolates was found to be a basic medium supplemented with sucrose at pH 7 ± 0.2 and a temperature of 28 ± 2°C. The formation of EPSs by rhizobacteria is one of the important mechanisms in exerting drought tolerance. Based on the qualitative results, it was found that environmental stresses such as pH and temperature stimulated the production of EPS. Higher EPS production has been indicated in culture media supplemented with sucrose and glucose [57]. These variations are due to the kinds of sugar used and enzymatic metabolism of each strain [58] and the activity of glucosyltransferases [59, 60]. EPS is fundamental for microbial life and provides an ideal environment for chemical reactions, nutrient entrapment, and protection against environmental stresses such as salinity and drought [61]. The EPS plays an important role in soil aggregation, thereby improving soil water holding capacity and soil fertility as observed in *Azospirillum* [15, 62]. Bacterial EPS production is one mechanism to survive under stressful (drought) conditions [63].

This study finds a wider range of NaCl tolerance although higher concentration brings a drastic decline in bacterial growth. It was observed that the extent to which growth was suppressed was directly proportional to the increasing concentration of NaCl. Therefore, some of the salt-tolerant isolates (in this study) had good saprophytic and competitive abilities to perform well in drought-stressed conditions. It seems that this high osmotic strength is due to the production of proline, glutamate, glycine, betaine, and trehalose in the cells. Na^+^ accumulation declines soil porosity, soil aeration, and water conductance. High Na^+^ ions also interfere with K^+^ and Ca^2+^ and affect enzymatic activities [64]. Soil bacteria inhabiting salty and arid ecosystems have the potential to promote plant growth under salinity and drought conditions [65]. Drought conditions are accompanied by an increase in temperature, changes in soil pH, heavy metals, and salinity. Therefore, the successful deployment of PGPR in stressed ecosystems depends on their ability to withstand and proliferates under adverse environments [66].

Seven isolates were identified with high-temperature (45°C) tolerance. Among tested strains, *Bacillus* spp. exhibited higher tolerance of temperature than *Pseudomonas* spp. One possible reason for this is due to the synthesis of heat-shock proteins [50] and also the presence of extremely resistant and dormant endospores produced by *Bacillus* spp. [67]. Similarly, the formation of endospores by *Bacillus* isolates could enhance their tolerance to high temperature [45]. Moreover, [67] highlighted that *Bacillus* endospores are extremely resistant and capable of withstanding unfavorable conditions. A thermotolerant *P. putida* NBRI0987 was isolated from the drought-affected rhizosphere of chickpea (*Cicer arietinum*) [68]. Another study has reported that a strain of *Pseudomonas* AKM-P6 possessing plant growth-promoting properties enhanced the tolerance of sorghum seedlings to high temperatures (47–50°C). Studies suggested that rhizobacterial isolates RR-1, GGP-1, and GNR-1 were both tolerant to high temperature (45°C) and also exhibited multiple beneficial plant growth-promoting activities [69]. Although *in vitro* temperature selection is not considered as a promising approach for field applications, but high temperature tolerance can be useful for isolating competitive PGPR in oscillating temperature in the fields [70].

In this study, four isolates *P. polymyxa* strain FB-50, *A. calcoaceticus* stain BS-27, *P. putida* strain BS-19, and *P. fluorescens* strain FB-49 showed 100% tolerance to all HMs tested. The result revealed that PGPR showed sensitivity to the different concentrations of HMs applied. The tolerance of *Paenibacillus* spp and *B*. *thuringiensis* to HMs like Cd, Cu, and Zn was also reported in [71]. *Pseudomonas* sp. showed a 97.9% Pb, 93.5% Cd, and 68% Cu removal efficiency from contaminated industrial wastewater is reported [72]. Surface binding/reduced uptake, increased efflux intracellular sequestration, enzyme detoxication, and active transport are among the proven mechanisms of tolerance [73]. Hence, these isolates could be useful in the bioremediation of HM-polluted environments.

The results of this study proved that the inoculation of PGPR isolates alone or in consortia had a significant effect in acacia growth under controlled conditions and also ameliorates the negative effect of drought stress. Inoculation with consortia showed the best plant growth performance and enhanced drought tolerance owing to their synergistic benefits in order to enhance plant growth. Similarly, inoculation with individual strains also improved plant biomass SH, RL, leave numbers, SDW, and RDW compared to the control. *P. fluorescens* strain FB-49 resulted in the highest biomass increase in under water restriction followed by *P. polymyxa* FB-50. Bacterial inoculation significantly increased the biomass of palm under drought, thereby contributing an essential ecological service to the entire oasis ecosystem. The possible mechanisms associated with PGPR-derived drought tolerance include alterations in host root system architecture, osmoregulation, management of oxidative stress, production of EPS, and transcriptional regulation of host stress response genes [47, 52, 74, 75]. Moreover, PGPR maintains the water budget of plants by improving the growth of the root system. This improves the water use efficiency and water absorption ability of roots under water scarcity [76]. Inoculation of plants with PGPR increases the growth rate/yield and fosters seedlings emergence in plants under greenhouse trials [77] and enhanced the root system (up to 40%) in pepper [78]. Also, in [79], it is reported that inoculation with *Enterobacter* spp and *Klebsiella* spp increased in the dry matter of *Lupinus albescens* by 75 and 81%, respectively, compared to the control. The application of *Bacillus subtilis* (BERA 71) turned out to be potentially beneficial in ameliorating the deleterious impact of salinity and drought in *Acacia gerrardii* [80]. Drought enhancement in *Sambucus williamsii* via the inoculation of *A. calcoaceticus* X128 was reported [81]. *P. polymyxa* enhanced the drought tolerance in *Arabidopsis thaliana* [82]. *Acinetobacter* spp and *Pseudomonas* spp enhanced the shoot and leaf biomasses of drought-challenged grapevines indicating the PGP activity of phytobeneficial microbes [83]. This study suggests the integrative use of a combination and/or single application of PGPR strains to be a promising and eco-friendly strategy for reducing moisture stress in plants.

## 5. Conclusion

This study revealed that PGPR strains recovered from degraded lands in Ethiopia have exhibited a promising abiotic stress-tolerance capacity. Some bacterial strains were considered completely tolerant (CT) to induced osmotic stress. Most of the bacterial isolates were biofilm formers and EPS producers which play protective roles under stressing conditions. Some PGPR strains such as *P. polymyxa*, *A. calcoaceticus*, *P. putida,* and *P. fluorescens* enhanced the drought stress tolerance in acacia under greenhouse conditions. Mixed inoculation resulted in higher drought tolerance in comparison to single inoculation. Thus, the elite indigenous strains identified in this study are potentially used in field trials to confirm their performance and applicability for the rehabilitation of degraded environments.

## Figures and Tables

**Table 1:** *In vitro* top drought stress-tolerant PGPR isolates and the closest species identity based on the 16S rRNA gene sequence analysis.

Isolate code	Closest relatives	Best match ID (NCBI)	Query cover (%)	% Similarity	Gene bank accessions
BS-45	*Bacillus thuringiensis*	CP021436.1	100	100	MN005965
PS-16	*Enterococcus gallinarum*	JF915769.1	99	99	MN005973
BS-51	*Paenibacillus polymyxa*	CP006872.1	98	100	MN005974
FB-50	*Paenibacillus polymyxa*	CP025957.1	100	100	MN005976
RS-79	*Agrobacterium tumefaciens*	CP033032.1	100	99	MN005977
RS-58	*Ochrobactrum intermedium*	KC146415.1	100	100	MN005978
RS-59	*Ochrobactrum intermedium*	AJ242582.2	92	99	MN005979
RS-66	*Ochrobactrum intermedium*	AJ242582.2	99	99	MN005982
RS-72	*Ochrobactrum intermedium*	KC146415.1	100	100	MN005988
BS-27	*Acinetobacter calcoaceticus*	KC257031.1	99	99	MN005992
BS-19	*Pseudomonas putida*	CP025262.1	99	99	MN005993
BS-26	*Pseudomonas plecoglossicida*	MF281997.1	100	99	MN005997
BS-44	*Pseudomonas fulva*	CP014025.1	100	99	MN006005
BS-53	*Pseudomonas fulva*	CP014025.1	100	99	MN006006
FB-49	*Pseudomonas fluorescens*	KY228953.1	100	100	MN006008
PS-2	*Klebsiella michiganensis*	CP033824.1	100	99	MN006010
PS-3	*Klebsiella oxytoca*	CP033824.1	99	99	MN006011
BS-46	*Morganella morganii*	CP032295.1	99	99	MN006012
PS-6	*Morganella morganii*	CP032295.1	100	99	MN006013
PS-14	*Morganella morganii*	CP032295.1	100	99	MN006017
RS-65	*Serratia marcescens*	CP021164.1	99	99	MN006026
RS-54	*Serratia fonticola*	LR134492.1	100	99	MN006030

**Table 2:** *In vitro* features of the strains selected based on water stress-tolerant potentials.

S. No.	Isolate code	Closest relatives	Mean ± SD 600 nm OD (*n* = 3)	Tolerance levels
1	BS-45	*Bacillus thuringiensis*	0.53 ± 0.12	CT
2	PS-16	*Enterococcus gallinarum*	0.54 ± 0.17	CT
3	FB-50	*Paenibacillus polymyxa*	0.51 ± 0.16	CT
4	RS-79	*Agrobacterium tumefaciens*	0.57 ± 0.20	CT
5	RS-72	*Ochrobactrum intermedium*	0.52 ± 0.22	CT
6	BS-27	*Acinetobacter calcoaceticus*	0.59 ± 0.19	CT
7	BS-19	*Pseudomonas putida*	0.60 ± 0.12	CT
8	FB-49	*Pseudomonas fluorescens*	0.64 ± 0.15	CT
9	PS-2	*Klebsiella michiganensis*	0.50 ± 0.17	CT
10	RS-65	*Serratia marcescens*	0.55 ± 0.15	CT

Values are mean ± standard deviation.

**Table 3:** Growth determined at OD 600 nm of selected PGPR isolates at varying pH ranges.

Isolate	pH4	pH5	pH7	pH9	pH10
BS-27	0.12 ± 0.01^c^	0.44 ± 0.04^cd^	1.26 ± 0.21^abcd^	0.68 ± 0.02^bc^	1.13 ± 0.09^abc^
FB-50	0.14 ± 0.02^bc^	0.43 ± 0.05^cd^	1.32 ± 0.19^abcd^	0.4 ± 0.08^def^	1.11 ± 0.12^abc^
RS-45	0.08 ± 0.01^c^	0.15 ± 0.05^e^	1.39 ± 0.09^ab^	0.3 ± 0.05^ef^	1.17 ± 0.11^ab^
FB-49	0.26 ± 0.04^a^	0.67 ± 0.09^bc^	1.46 ± 0.11^a^	0.85 ± 0.14^ab^	1.32 ± 0.23^a^
RS-72	0.12 ± 0.04^c^	0.24 ± 0.05^de^	1.05 ± 0.04^b^	0.42 ± 0.09^ef^	0.97 ± 0.07^bcd^
BS-19	0.11 ± 0.02^c^	0.14 ± 0.06^e^	1.11 ± 0.10^bcd^	0.26 ± 0.06^f^	0.80 ± 0.11^cd^
RS-79	0.21 ± 0.02^ab^	0.84 ± 0.06^a^	1.20 ± 0.14^abcd^	0.51 ± 0.15^cde^	0.70 ± 0.15^d^
PS-16	0.07 ± 0.02^c^	0.42 ± 0.05^cd^	1.39 ± 0.06^ab^	0.54 ± 0.14^cde^	1.13 ± 0.116^abc^
BS-65	0.09 ± 0.02^c^	0.63 ± 0.05^b^	1.09 ± 0.10^cd^	0.65 ± 0.15^bcd^	1.01 ± 0.04a^bcd^
PS-2	0.06 ± 0.03^c^	0.49 ± 0.08^bc^	1.34 ± 0.03^abc^	0.93 ± 0.05^a^	1.18 ± 0.03^ab^

Means with the same letter down the column are not significantly different with mean ± SD, *n* = 3.

**Table 4:** Effect of temperature on the growth of selected PGPR isolates OD readings at 600 nm.

Isolates	Temp. 15°C	Temp. 25°C	Temp. 35°C	Temp. 40°C	Temp. 45°C
BS-27	0.30 ± 0.04^a^	0.89 ± 0.07^ab^	1.13 ± 0.04^abc^	0.89 ± 0.14^abc^	0.55 ± 0.05^b^
FB-50	0.25 ± 0.07^abc^	0.98 ± 0.11^ab^	1.16 ± 0.02^abc^	0.82 ± 0.15^bc^	1.01 ± 0.09^a^
RS-45	0.19 ± 0.04^abc^	0.85 ± 0.17^ab^	0.87 ± 0.05^bc^	1.12 ± 0.02^abc^	0.95 ± 0.11^a^
FB-49	0.26 ± 0.05^ab^	1.21 ± 0.14^a^	1.36 ± 0.19^ab^	1.10 ± 0.12^a^	0.94 ± 0.14^a^
RS-72	0.16 ± 0.01^bc^	0.76 ± 0.05^b^	1.05 ± 0.02^c^	0.73 ± 0.03^c^	0.29 ± 0.03^b^
BS-19	0.22 ± 0.02^abc^	0.93 ± 0.07^ab^	1.39 ± 0.09^a^	0.85 ± 0.07^abc^	0.53 ± 0.05^b^
RS-79	0.21 ± 0.04^abc^	0.80 ± 0.13^b^	1.29 ± 0.07^abc^	0.73 ± 0.05^c^	0.55 ± 0.03^b^
PS-16	0.17 ± 0.02^abc^	0.98 ± 0.09^ab^	1.13 ± 0.07^abc^	0.92 ± 0.10^abc^	0.45 ± 0.07^b^
BS-65	0.21 ± 0.03^abc^	0.90 ± 0.11^ab^	1.15 ± 0.09^abc^	0.72 ± 0.09^c^	0.54 ± 0.04^b^
PS-2	0.12 ± 0.02^c^	0.75 ± 0.06^b^	1.14 ± 0.07^abc^	1.07 ± 0.06^ab^	0.47 ± 0.09^b^

Means with the same letter down the column are not significantly different. Mean ± SD of three replicates using Duncan's multiple range test (*p* ≤ 0.05) *n* = 3. Temp = temperature.

**Table 5:** Heavy metal tolerance profile of ten potential PGPR strains at a varying concentration of HMs.

S. No.	Isolates	Tolerance (*μ*g/ml)															
		Fe 50	Fe 100	Fe 300	Mn 50	Mn 100	Mn 300	Cu 50	Cu 100	Cu 300	Zn 50	Zn 100	Zn 300	Pb 50	Pb 100	Pb 300	**% (R)**
1	*Bacillus thuringiensis* BS-45	T	T	T	T	T	S	T	T	T	T	T	T	T	T	T	**90**
2	*Enterococcus gallinarum* PS-16	T	T	T	T	T	T	T	S	S	T	T	S	T	T	T	**70**
3	*Paenibacillus polymyxa* FB-50	T	T	T	T	T	T	T	T	T	T	T	T	T	T	T	**100**
4	*Agrobacterium tumefaciens* RS-79	T	T	T	T	T	T	T	T	S	T	T	S	T	T	T	**80**
5	*Ochrobactrum intermedium* RS-72	T	T	T	T	T	S	T	T	T	T	S	S	T	S	S	**50**
6	*Acinetobacter calcoaceticus* BS-27	T	T	T	T	T	T	T	T	T	T	T	T	T	T	T	**100**
7	*Pseudomonas putida* BS-19	T	T	T	T	T	T	T	T	T	T	T	T	T	T	T	**100**
8	*Pseudomonas fluorescens* FB-49	T	T	T	T	T	T	T	T	T	T	T	T	T	T	T	**100**
9	*Klebsiella michiganensis* PS-2	T	T	T	T	T	S	T	T	T	T	S	S	T	T	T	**70**
10	*Serratia marcescens* RS-65	T	T	T	T	T	S	T	S	S	T	S	S	T	T	T	**50**
	% (T)	**100**	**100**	**100**	**100**	**100**	**70**	**100**	**80**	**70**	**100**	**70**	**60**	**100**	**90**	**90**	
	% (S)	**0**	**0**	**0**		**0**	**30**	**0**	**20**	**30**	**0**	**30**	**40**	**0**	**10**	**10**	

T = tolerant and S = sensitive.

**Table 6:** Plant growth promotion in *A. abyssinica* treated with different PGPRs individually and consortium under control and drought stress conditions.

Treatment	SH (cm)/pot	RL (cm)/pot	No. of leaves/pot	SDW (g)/pot	RDW (g)/pot
Control	38.3 ± 2.9^i^	10.0 ± 2.6^i-k^	14.0 ± 2.6^f-i^	0.9 ± 0.2^jk^	0.12 ± 0.01^jk^
Control + D	31.7 ± 2.1^j^	7.0 ± 2.6^k^	9.3 ± 1.5^j^	0.6 ± 0.1^k^	0.1 ± 0.02^k^
BS-27	50.7 ± 0.6^d^	14.0 ± 2^f-h^	17.3 ± 1.2^c-e^	1.9 ± 0.4^b-e^	0.4 ± 0.06^d-i^
BS-27 +D	43.3 ± 1.5^f-h^	11.0 ± 1^h-j^	11.0 ± 2^ij^	1.5 ± 0.2^e-i^	0.3 ± 0.04^g-j^
FB-50	52.0 ± 2.6^d^	17.7 ± 1.5^b-e^	18.0 ± 3^b-e^	1.9 ± 0.3^b-e^	0.4 ± 0.11^d-i^
FB-50 +D	46.3 ± 3.1^ef^	11.7 ± 2.1^h-j^	15.7 ± 2.5^d-g^	1.7 ± 0.1^g-j^	0.5 ± 0.05^g-i^
RS-65	46.7 ± 3.2^ef^	16.0 ± 1^d-f^	21.3 ± 3.1^ab^	1.5 ± 0.4^e-i^	0.3 ± 0.09^e-i^
RS-65 +D	39.3 ± 2.5^hi^	11.0 ± 2^h-j^	18.0 ± 2^b-e^	1.1 ± 0.2^ij^	0.2 ± 0.05^j-k^
FB-49	61.0 ± 1.7^b^	20.3 ± 1.5^ab^	21.3 ± 2.1^ab^	2.2 ± 0.4^bc^	0.60.03^bc^
FB-49 +D	51.7 ± 1.5^d^	18.7 ± 1.5^b-d^	17.7 ± 1.5^c-e^	1.8 ± 0.1^d-h^	0.55 ± 0.07^c-f^
RS-72	51.7 ± 2.1^d^	16.7 ± 1.5^c-f^	18.3 ± 2.5^b-d^	2.1 ± 0.4^b-d^	0.5 ± 0.12^cd^
RS-72 +D	44.0 ± 2^fg^	11.3 ± 1.5^h-j^	13.7 ± 1.5^f-i^	1.3 ± 0.1^g-i^	0.4 ± 0.1^e-h^
BS-19	48.3 ± 1.5^de^	14.0 ± 1^f-h^	18.7 ± 3.1^b-d^	2.2 ± 0.3^bc^	0.5 ± 0.14^c-e^
BS-19 +D	40.3 ± 2.5^g-i^	10.3 ± 1.5^ij^	13.0 ± 2^j-i^	1.6 ± 0.3^d-h^	0.4 ± 0.04^d-f^
RS-79	43.3 ± 2.5^f-h^	12.7 ± 2.1^g-i^	15.3 ± 1.5^d-g^	1.9 ± 0.3^c-f^	0.4 ± 0.11^d-h^
RS-79 +D	39.3 ± 1.5^hi^	8.7 ± 1.5^jk^	11.3 ± 1.5^i-j^	1.4 ± 0.2^f-i^	0.3 ± 0.08^g-j^
PS-16	46.0 ± 2^ef^	11.0 ± 1^h-j^	20.3 ± 2.5^bc^	1.8 ± 0.5^c-f^	0.3 ± 0.12^g-i^
PS-16 +D	39.7 ± 2.5^hi^	8.4 ± 2.1^jk^	15.7 ± 2.5^d-g^	1.4 ± 0.2^g-i^	0.2 ± 0.06^g-j^
BS-45	51.0 ± 2^d^	11.3 ± 1.5^h-j^	14.7 ± 1.5^e-h^	2.4 ± 0.2^b^	0.4 ± 0.15^d-i^
BS-45 +D	42.7 ± 2.1^f-h^	9.3 ± 1.5^i-k^	11.0 ± 1^ij^	1.4 ± 0.1^g-i^	0.2 ± 0.08^h-k^
PS-2	41.3 ± 1.5^g-i^	10.0 ± 1^i-k^	18.3 ± 1.5^b-d^	1.4 ± 0.1^g-i^	0.2 ± 0.07^g-j^
PS-2 +D	38.7 ± 2.1^i^	8.3 ± 1.5^jk^	11.7 ± 2.1^h-j^	1.2 ± 0.1^h-i^	0.2 ± 0.02^i-k^
Consortia	70.3 ± 1.5^a^	22.3 ± 2.1^a^	23.7 ± 1.5^a^	2.9 ± 0.3^a^	0.9 ± 0.12^a^
Consortia +D	57.3 ± 1.5^c^	19.3 ± 2.5^bc^	16.7 ± 2.8^d-f^	2.1 ± 0.2^b-d^	0.8 ± 0.07^ab^

Means with the same letter down the column are not significantly different at (*p* ≤ 0.05) by using DMRT. Mean ± SD (*n* = 3); *D* represents drought. Consortia = FB-50 + BS-27 + BS-19 + FB-49. SH = shoot height, RL = root length, SDW = shoot dry weight, RDW = root dry weight.

## Data Availability

The data used to support the study are included within the manuscript. Data with all replicates can be made available from the corresponding author upon request.

## Supplementary Materials

Supplementary Materials Supplementary Figure S1: detection of biofilm production by Congo red agar (CRA) method. Supplementary Figure S2: detection of biofilm production by the tube method (TM) Supplementary Figure S3: translucent colony, potentially producing EPS, and control without EPS production.
