# Supplementary material for: Plant Growth-Promoting Rhizobacteria Isolated from Degraded Habitat Enhance Drought Tolerance of Acacia (Acacia abyssinica Hochst. ex Benth.) Seedlings
Source: Int J Microbiol. 2020 Oct 29;2020:8897998. doi: 10.1155/2020/8897998 (PMC7646561; doi:10.1155/2020/8897998)
Supplement: Supplementary Materials — Supplementary Figure S1: detection of biofilm production by Congo red agar (CRA) method. Supplementary Figure S2: detection of biofilm production by the tube method (TM) Supplementary Figure S3: translucent colony, potentially producing EPS, and control without EPS production. [file 8897998.f1.docx]

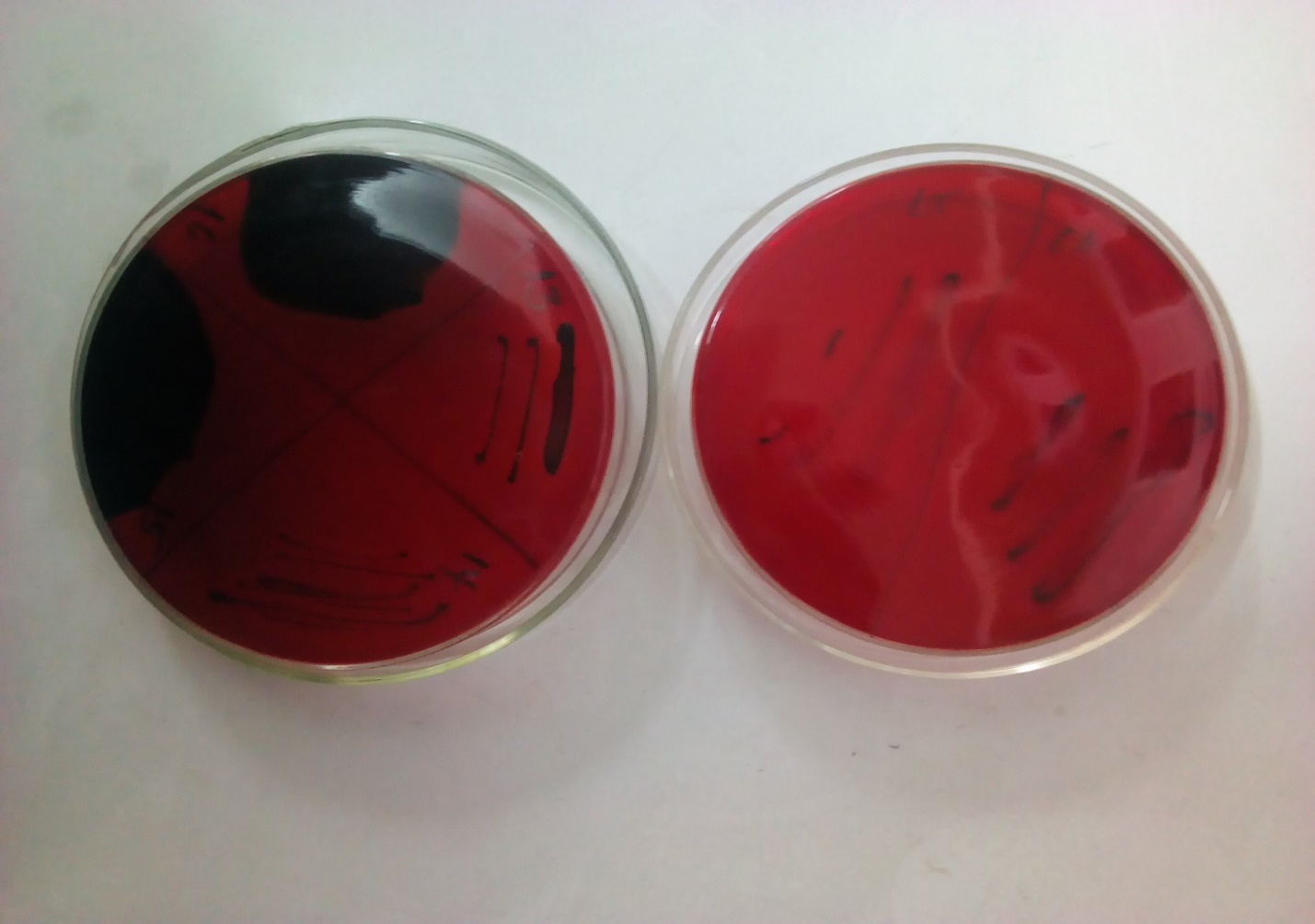


Strong Biofilm Formation

Weak Biofilm formation

Moderate Biofilm Formation

Supplementary Figure S1. Detection of biofilm production by Congo red agar (CRA) method


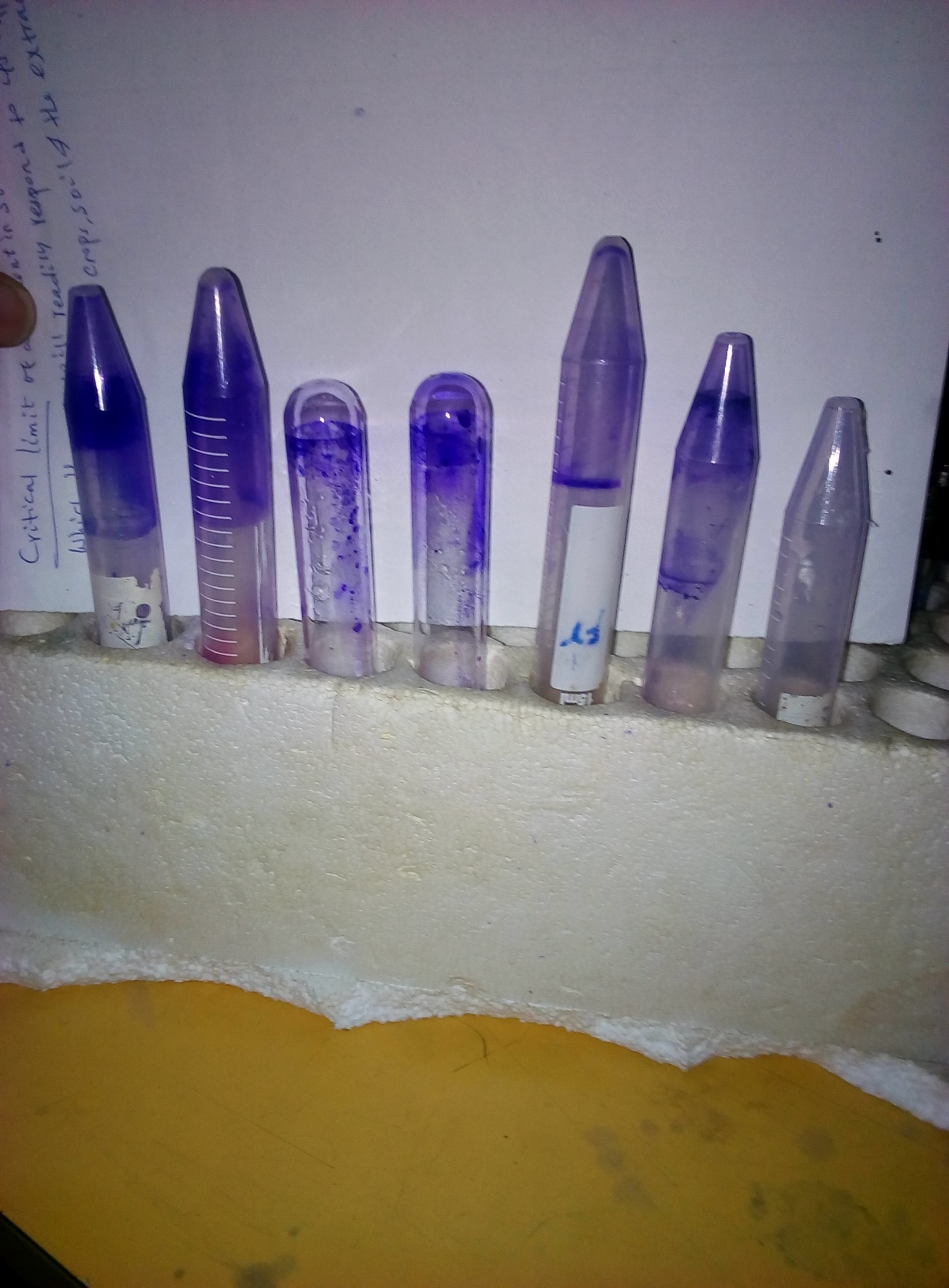


Control

Biofilm ring formation on TM

Supplementary Figure S2. Detection of biofilm production by tube method (TM)

EPS Producing

Control


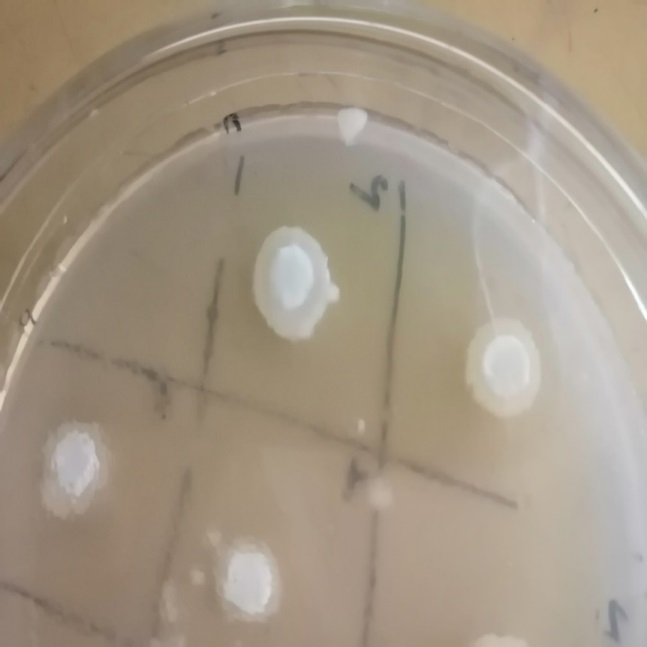

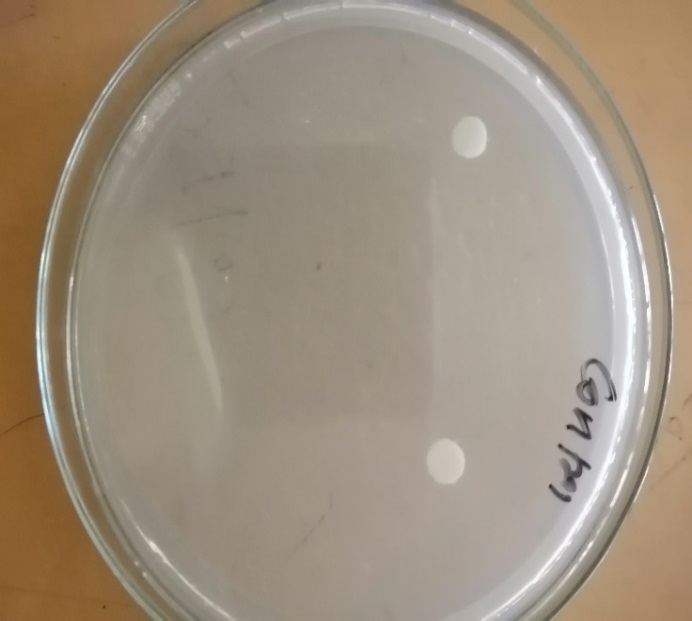


EPS Positive (mucoid colonies)

Control (EPS Negative)

Supplementary Figure S3. Translucent colony, potentially producing EPS and control without EPS production.


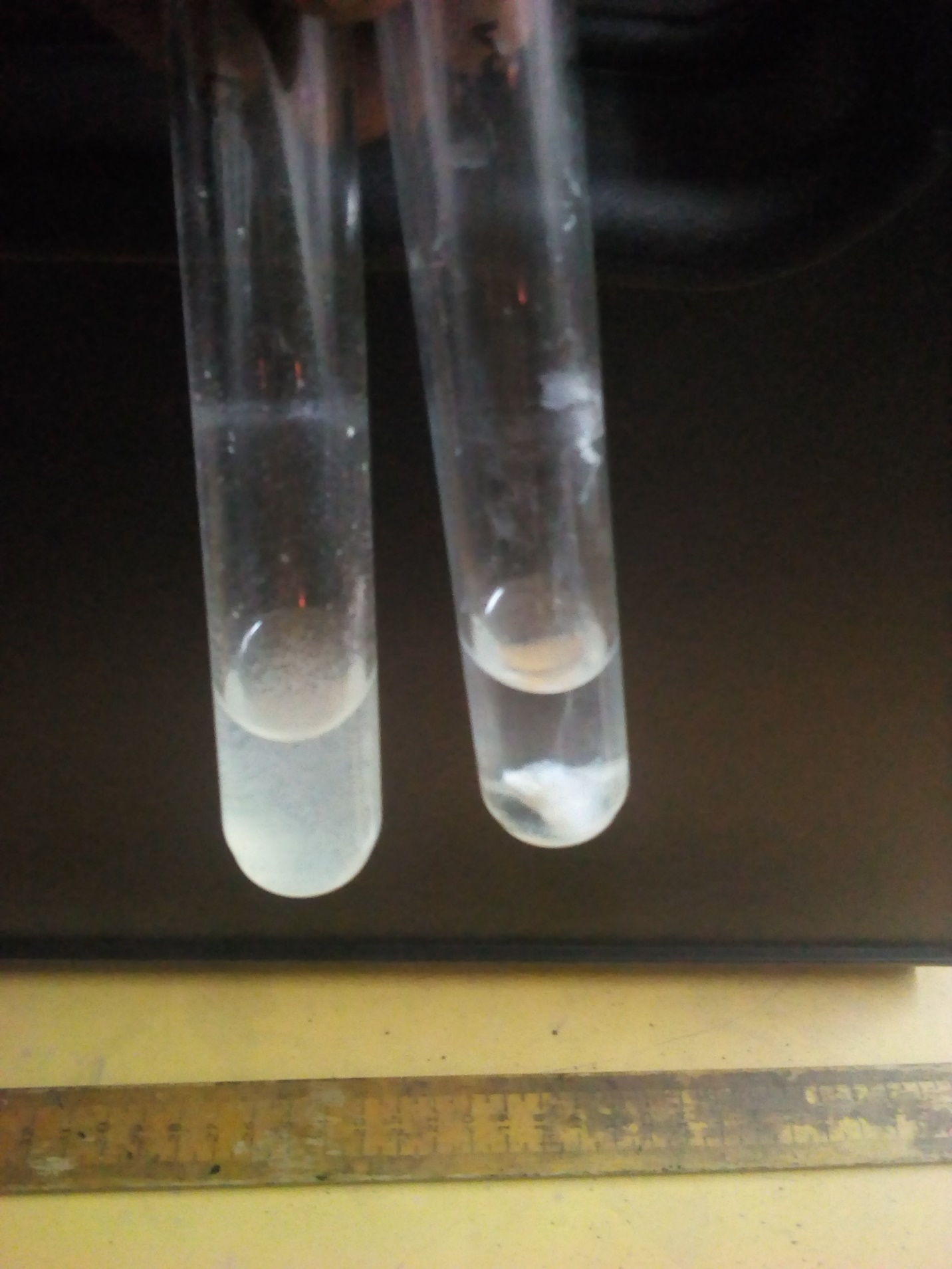


EPS (precipitate formation)

Cell Mass with no EPS

Ethanol

Supplementary Figure S4. Confirmation of EPS production by mixture of colonies in ethanol
